# Inhibition of secretory leukocyte protease inhibitor (SLPI) promotes the PUMA-mediated apoptosis and chemosensitivity to cisplatin in colorectal cancer cells

**DOI:** 10.1007/s12672-022-00535-9

**Published:** 2023-01-03

**Authors:** Zhijiang Wei, Guiying Liu, Rufu Jia, Wei Zhang, Li Li, Yuanyuan Zhang, Zhijing Wang, Xiyong Bai

**Affiliations:** 1 The First Department of Tumor Surgery, Cangzhou Central Hospital, Cangzhou, 061001 Hebei People’s Republic of China; 2 The Brain Science Hospital of CangZhou Central Hospital, Cangzhou, 061001 Hebei People’s Republic of China

**Keywords:** Secretory leukocyte protease inhibitor, Colorectal cancer, PUMA, Cisplatin, NF-κB signaling, Chemosensitivity

## Abstract

**Background:**

Aberrant expression of Secretory Leukocyte Protease Inhibitor (SLPI) has been associated with human cancer growth and its suppression was identified as a potential target for anti-cancer drugs, particularly in colorectal cancer. However, the underlying mechanism by which SLPI affected the development of drug resistance in CRC remains unclear.

**Objective:**

This study investigated the role of SLPI in the p53-up-regulated modulator of apoptosis (PUMA)-mediated CRC cells’ apoptosis and their chemosensitivity to Cisplatin.

**Methods:**

A series of qRT-PCR and western blot analyses were performed to characterize the expressions of SLPI, PUMA, and Akt in CRC lines. Tunel, transwell, and CCK-8 analyses were monitored to define the impacts of the siRNA-mediated knockdown of SLPI on CRC cell development. Furthermore, in vivo development of CRC was evaluated in nude mice infected with siSLPI or Cisplatin alone or both, and Ki67 and caspase-3 immunohistochemistry assay was monitored on multiple tissue microarray from the same cohort.

**Results:**

Our results showed that SLPI inhibition strongly promoted the expressions of the pro-apoptotic protein PUMA, cleaved-caspase3 and Bax and reduced the cell viability of HT29 and HT116 cell lines in vitro. In addition, siSLPI knockdown effectively suppressed both Akt and FoxO3 proteins and improved the sensitivity to cisplatin chemotherapy. Xenograft tumor assay revealed a lowered growth in mice treated with Cisplatin, while combined treatment of siSLPI achieved more significant anticancer effects than Cisplatin alone.

**Conclusions:**

Taken together, these findings demonstrated that suppression of SLPI might repress the growth of human colorectal cancer cells both in vitro and in vivo*.* These results suggested SLPI as a novel resistance factor to Cisplatin, and a combination of Cisplatin and SLPI inhibitor be beneficial for colorectal cancer therapy.

**Supplementary Information:**

The online version contains supplementary material available at 10.1007/s12672-022-00535-9.

## Introduction

Colorectal cancer (CRC) is one of the most ubiquitous malignancies and the second leading cause of cancer-related mortality on the planet, and it is among the most lethal along with breast, lung, and prostate cancers. [1, 2]. The survival rates among cancer patients have been considerably lessened due to the screening and the evolution of cancer therapies such as surgery and chemotherapy, notably [3, 4]. The survival among colon cancer patients has augmented throughout the recent years; around 40% ± 10% of patients experienced sickness weakening or metastasis [5].

Recent studies have identified many genes that play pivotal roles in various cancers; the secretory leukocyte protease inhibitor (SLPI), mainly generated and released by epithelial and inflammatory cells, has been widely reported as an emerging anti-inflammatory. SLPI represents a chain protein with 11.7 kDa of protein weight, non-glycosylated, and expressed in various cell types such as lung epithelial cells, secretory cells of the salivary glands, and other inflammatory hosts [6–8]. Recent investigations have demonstrated that aberrant expression of SLPI was strictly linked to various human cancers development like lung, ovarian, cervix, neck, and pancreas cancers [9–11]. In addition, the expression of SLPI was associated with different stages of tumor development, including the stages of TNM (tumor, node, metastasis), lymph node metastasis, and distant metastasis. A recent report showed that the overexpression of SLPI in colon cancer tissues could be associated with severe pathological characteristics and suggested its potential role in colorectal cancer cell prognosis [12]. Also, the overexpression of the SLPI gene from mammary tumor cells reduced tumor development and enhanced the survival rate among mice [13] and was thought to exert pro-apoptotic functions and suppress tumor cell growth [14, 15]. To the best of our knowledge, the present study discussed for the first time the roles of SLPI in the chemosensitivity of colon cancer cells.

Currently, the only potential mechanism revealed in the regulatory pathway of SLPI remains the NF-κB signaling pathway which plays an essential role in modulating inflammatory responses, cell development, differentiation, and apoptosis. In addition, NF-κB was reported to mediate the activation of PUMA proteins in response to Cabozantinib in colorectal cancer cells [17]. At the same time, the PUMA proteins are reputed to generate apoptosis and are associated with the Bcl-2 BH3-only family, an excellent stimulator of the mitochondrial membrane permeability [18]. The interruption of p53-dependent PUMA directly affects the viability of cancer cells and their chemotherapeutic resistance, while transcription factors such as p6521, FoxO3a20, p7323, and CHOP22 were also associated with PUMA initiation independent of p53 reactions [19]. The Akt inhibition promoted the p53-independent PUMA apoptosis through the synchronized activation of FoxO3a and NF-κB, two molecules that can provoke the Bax-mediated intrinsic mitochondrial apoptosis. Previous studies have reported that the PUMA-induced apoptosis in colon cancer was regulated by either the Akt/FoxO3a/PUMA signaling pathway (the principal pathway) or Akt/NF-κB/PUMA accessory pathway [16]. The PI3K/Akt signaling pathway is widely studied in human cancers because of its pivotal role in cell survival. Protein kinase B (Akt) represents the critical molecule of crosstalk between the PI3K/Akt signaling pathway and many other cells' signaling networks involved in crucial cell metabolisms such as cell growth and division and inhibition and apoptosis. Many studies have also associated the dysfunction of the Akt signaling pathway to several chemotherapy drugs resistances, including in prostate, ovarian, pancreatic, and breast cancers [17–19].

Therefore, this study targeted the SLPI inhibitory effects on PUMA Akt and NF-κB protein expression and highlighted its repercussions on developing CRCs and chemosensitivity to cisplatin drugs. The suppression of SLPI induced remarkable changes in the expressions of both caspase-3 and Bax proteins, causing caspase-3-inducted apoptosis in colon cancer. It aimed to probe the effects of SLPI suppression on HT29 and HT116 colorectal cancer cell apoptosis and chemosensitivity. Finally, the results demonstrated that SLPI targeted Akt/NF-κB/PUMA axis to regulate the development and chemosensitivity of colon cancer cells.

## Material and methods

### Cell culture

All cells were taken out from the refrigerator at − 80 ℃ and quickly thawed in a water bath at 37 ℃ for 2 min. Then, cells were centrifuged at 1000 r/min for 5 min. At the same time, 5 mL of DMEM complete medium (DMEM basic medium + 10% foetal bovine serum + 1% double-antibody made of penicillin (10,000 IU) and streptomycin (10,000 μg/mL) 100 * working solution) was added to a 60 mm dish. After centrifugation, 1 mL of complete medium was taken to resuspend the cells until ready. Cells were cultured overnight in a 5% carbon dioxide incubator at 37 ℃.

### Cell transfection

HT29 and HCT116 cells were cultured in 6-well plates (1.0 × 105) for 24 h, then transfected and separated into four groups, including those treated with empty vector (NC) and the siSLPI group (transfected with the small interfering RNA or siRNA plasmid). The silenced SLPI (siSLPI) and the empty vector control were obtained from RiboBio (Guangzhou, China). Then, the stable transformation was applied using Lipofectamine 2000 (Invitrogen, SanMateo, CA, USA) by following the manufacturer's protocol. The total of 20 µM of siRNA and NC supplemented with 5 µL Lipofectamine 2000 was added to Opti-MEM^®^ alighted medium and incubated at 25 ℃ for 10 min. Finally, Lipofectamine 2000 was merged with each sample and grown in a fresh-made Opti-MEM^®^ RPMI 1640 medium for 6 h in culture before putting back to 10% FBS RPMI 1640 medium.

### Cell proliferation/viability assay

When the confluency of the cells reached 80%, we added cisplatin drugs accordingly and cultured them in a 5% CO_2_ incubator at 37 ℃ for 24–48 h. Add 10 μL CCK8 working solution to each well and continue to culture for 0.5 h. At 450 nm, each well’s absorbance value (OD450) was calculated, and the growth inhibition rate of each group of cells was calculated. Calculation formula: cell proliferation inhibition rate = (1 − absorbance value of experimental group/absorbance value of control group) × 100%, cell proliferation rate = (absorbance value of experimental group/absorbance value of control group − 1) × 100%. Each assay was applied in triplicate and cell proliferation rate is represented as fold change ± SEM in comparison to data of the first day.

### Western blot

The complete protein was extracted and quantified using the BCA method. We used the SDS-PAGE to collect 40 µg of total protein and transferred it to a PVDF membrane (GE Healthcare). Therefore, the membrane sealing, and antibody incubation were conducted with 5% skimmed milk powder at room temperature for 1 h. Subsequently, the antibodies were diluted into the blocking solution following the manuscript instructions and incubated with the membrane at 5 ℃ overnight. The primary antibodies included β—actin first antibody (beyotime), SLPI first antibody (Abcam, ab17157), PUMA first antibody (Abcam, ab9643) and p-foxo3 α first antibody (Abcam, ab9643), Ab47285) FoxO3 α first antibody (Abcam, ab12162) p65 (Abcam, ab16502) p-p65 (Abcam, ab76302) p-Akt (Abcam, ab81283) Akt (Abcam, ab8805) Bax (Abcam, ab32503) c-caspase3 (Abcam, ab2302) Ki67 (Abcam, ab15580). Furthermore, we used the TBST to wash the membrane incubated with a primary antibody three times, 10 min each time. Then, secondary antibody second antibody (beyotime) was diluted and incubated with the membrane at room temperature for 2 h, washed with TBST three times, 10 min each time before the bands' observation through Amersham ECL solution, and assessed with Multi gauge computer software (Berthold, Bundoora, Australia).

### Quantitative real-time PCR (qRT-PCR)

Total RNA was extracted from colorectal normal and siSLPI-transfected HT29 and HCT116 cells using TRIzol reagent, and through an ultraviolet spectrophotometer, we verified the RNA quality. Then, we performed reverse transcription to synthesize the cDNA from 1.0 µg of total RNA using a reverse transcription kit (Fermentas China Co., Ltd, Beijing, China). The qPCR analysis was carried out with the SYBR Green master kit (Applied Biosystems, USA). Therefore, we utilized 1 µL cDNA 10 µL solution as the template. The SLPI and PUMA primers were synthesized by Shanghai Shandon company according to the following sequences SLPI Forward primer 5′GAGATGTTGTCCTGACACTTGTG SLPI3′ Reverse 5′AGGCTTCCTCCTTGTTGGGT 5′ PUMA Forward primer 5′GACCTCA-ACGCACAGTACGAG PUMA5′ Reverse primer 5′AGGAGTCCCATGATGAGATTGT3′. The qPCR was conducted Following the 95 ℃ pre-denaturation for 30 s, 40 cycles of 95 ℃ for 30 s, and 60 ℃ of annealing temperature as per the thermocycler protocol. All samples were assessed three times. Relative expression of both PUMA and SLPI genes were evaluated through the ΔΔ Ct method, and the expression level was calculated as 2^−ΔΔCt^. Each value was normalized against that of GAPDH mRNA.

### Tunel

We used 4% paraformaldehyde for 30 min to fix all cells during the Tunel staining and ethanol and PBS for the clean-up. After the cell fixation and cleaning, PBS containing 0.3% Triton-X 100 was incubated at room temperature for 5 min to break the membrane and washed twice with PBS. Then, we added 100 μL TUNEL detection solution (biyuntian, c1086) to each sample, stained the solution overnight, and rewashed it with PBS 3 times. After fixing the sealing solution, we observed and took pictures using a fluorescence microscope.

### Immunofluorescence analysis

The culture medium was sucked out and washed with PBS 2–3 times, standing for 5 min each time; then fixed with 4% paraformaldehyde for 20 min and three times washed with PBS. Therefore, cells were treated with 0.5% Triton × 100 (in 1 * PBS) for 20 min, and PBS was used to clean three times, each time standing for 5 min. Finally, evaluate the subcellular localization of p-Akt, p-fox3a, and p-p65 in colon cancer cells evaluated following DAPI staining. HT-29 and HCT116 colorectal cells were treated with antibodies for 24 h, cleaned with PBS (pH 7.4), fixed with cold 70% ethanol, resuspended in DAPI, and incubated for 15 min at 37 °C wrapped in aluminium foil. The cells were then washed with PBS and examined under a Nikon Eclipse Fluorescence microscope (Nikon Instruments Inc., NY, USA).

### Animal husbandry

The animal experiments were approved by CangZhou Central Hospital Ethical Committee and the whole protocol was performed in accordance with institutional guidelines [20]. BALB/C nude mice (6–8 weeks) were reared in an IVC system and exposed to 12 h of light every day for 12 h at night. HT29 and HT116 cell lines were first transfected with siSLPI and then treated with Cisplatin after 24 h for chemotherapeutic treatments. The concentration of 1 × 10^6^ HT29 or HT116 cell lines was subcutaneously injected to each nude mouse for in vivo experiments. Four days after the tumor inoculation, siRNAs targeting SLPI were injected near the tumor, while the cisplatin was injected intraperitoneally at a dosage of 4 mg/kg body weight for each animal. Both siRNAs targeting SLPI and Cisplatin were injected twice a week for 2 weeks. The maximal tumour size/burden was not exceeded the permissible tolerance in institutional guidelines.

### Statistics

The whole data was reported as mean ± SD and assessed using SPSS 13.0 (SPSS, Chicago, IL). One-way ANOVA guided by Tukey’s test was applied to verify group variability. Unpaired Student’s t-test was applied to assess the differences between two groups for cell experiments We considered all differences as statistically significant with p < 0.05.

## Results

### SLPI plays a vital role in colorectal cancer cells

**Fig. 1:**
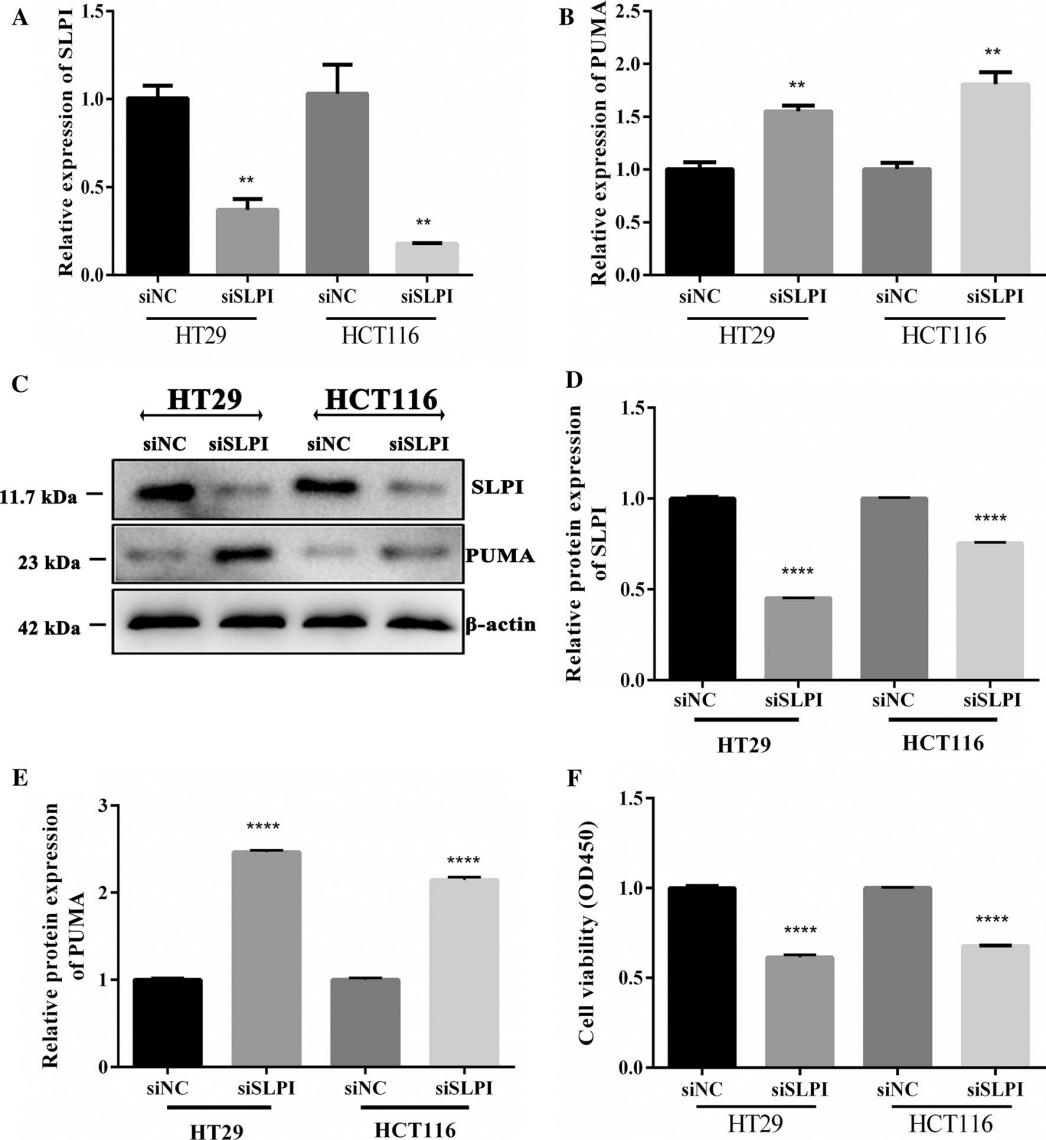
siSLPI reduced cell proliferation and enhanced PUMA activities. RT-PCR results showed the effective inhibition of siSLPI knockdown and PUMA's relative expression (**A**, **B**). WB showed that inhibition of SLPI was generated an increased expression of PUMA protein (**C**–**E**). Cell proliferation experiment showed that siSLPI significantly reduced the viability of both HT29 and HCT116 cells (**F**). *p < 0.05; ***p < 0.1, and ****p < 0.5

To further understand the inhibitory effects of SLPI on CRC cell development, human colorectal normal Cells (NC), a human colorectal adenocarcinoma cell line with epithelial morphology (HT29), and colon cancer cell line (HCT116) were all investigated under identical conditions. The qRT-PCR results showed that the suppression of SLPI promoted the expression of PUMA in both HT29 and HCT116 treated cells compared to NC (Fig. 1A, B). Interestingly, Western blot results were consistent with the mRNA, and PUMA’s protein level was more elevated in HT29 cancer cell lines after the siRNA knockdown (Fig. 1C–E). In addition, the silencing of SLPI led to a diminished viability rate in both HT29 and HCT116 cancer cells compared to NC (Fig. 1F). These results suggested SLPI as the regulator of PUMA activities in colorectal cancer cells in vitro.

### Inhibition of SLPI supressed the expressions of Akt and fox3a and promote the expressions of PUMA and p65 in colorectal cancer cells.

**Fig. 2:**
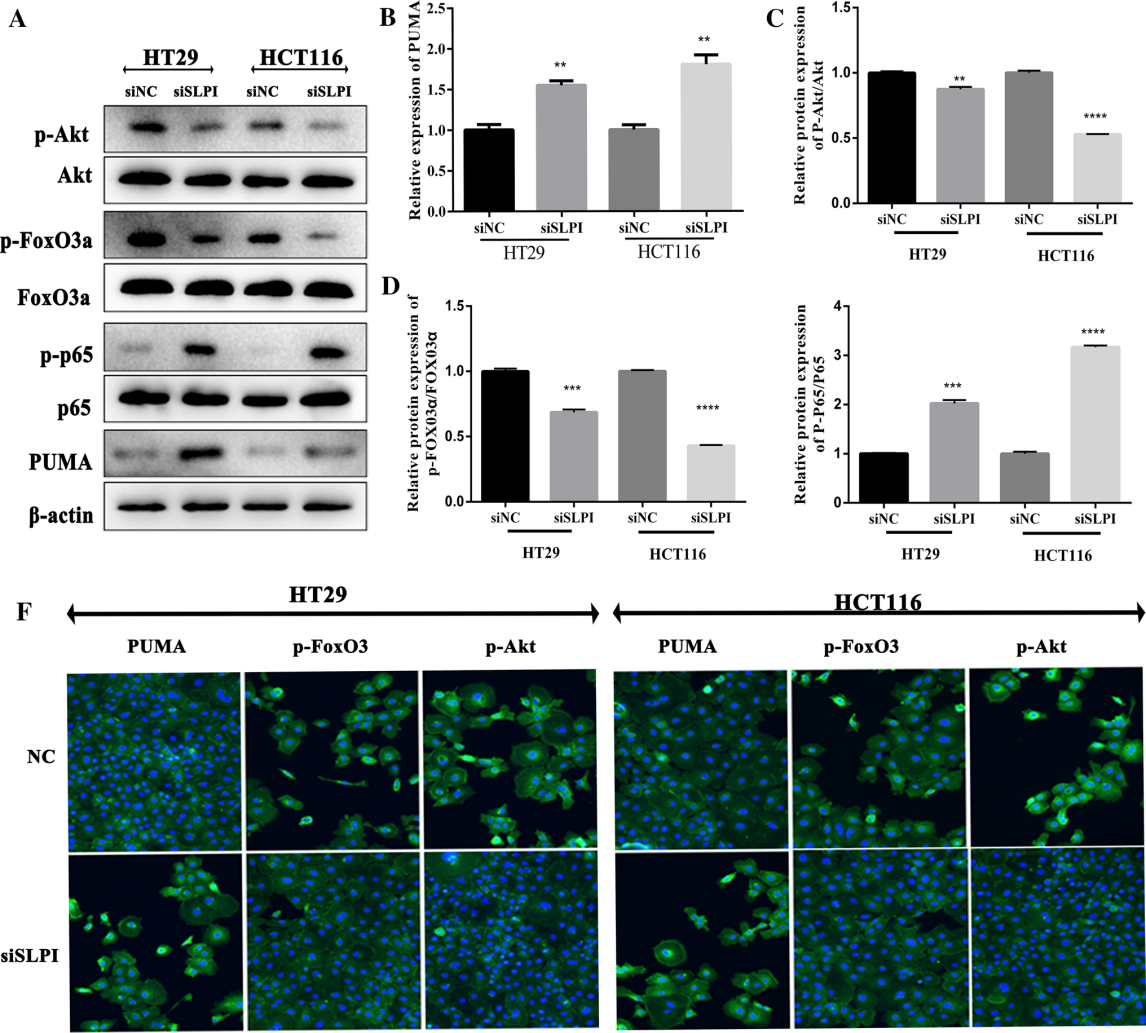
Inhibition of SLPI promotes PUMA expression through Akt related signaling pathway. **A** Western blot displaying how inhibition of SLPI decreased phosphorylation of both Akt p-fox3a proteins, while the phosphorylation of p65 was greatly increased. Scale bar representation of the protein expressions of Akt, FoxO3 and p65, respectively (**B**–**E**). Immunofluorescence results depicted the decreased levels of p-Akt and p-fox3a and upgraded levels of p-p65 in response to SLPI inhibition (**F**). *p < 0.05; ***p < 0.1, and ****p < 0.5

To understand the mechanism by which SLPI promoted the protein expression of PUMA in CRC, we investigated the expressions of three essential proteins (Akt, fox3a, and p65) in colorectal cancer cells under siSLPI treatments. Akt kinases are reputed for their crucial roles in cell survival, proliferation, and metastasis, while p65 plays a critical role in regulating the regulation of the NF-κB pathway. A previous study has revealed the crosstalk between Akt activation and the expression of SLPI in colon cancer [20]. Here, our western blot results showed that suppressing SLPI induced the reduction of the protein expressions of both Akt and fox3a in HT-29 and HCT116 lines, while the expression of the p-p65 protein was massively increased (Fig. 2A–E). Then, we applied the immunofluorescence assay to visualize and confirm the immune checkpoint. We found that the SLPI knockdown slowed down the movement of p-Akt and p-fox3a but enhanced the translocation of p-p65 into the CRC nucleus (Fig. 2F). Thus, these results confirmed that SLPI is the downstream regulator of Akt, fox3a, and p65 protein expressions and movement within the cells.

### SLPI inhibition promotes the CRC cell apoptosis by activating PUMA/BAX pathway in CRCs

**Fig. 3:**
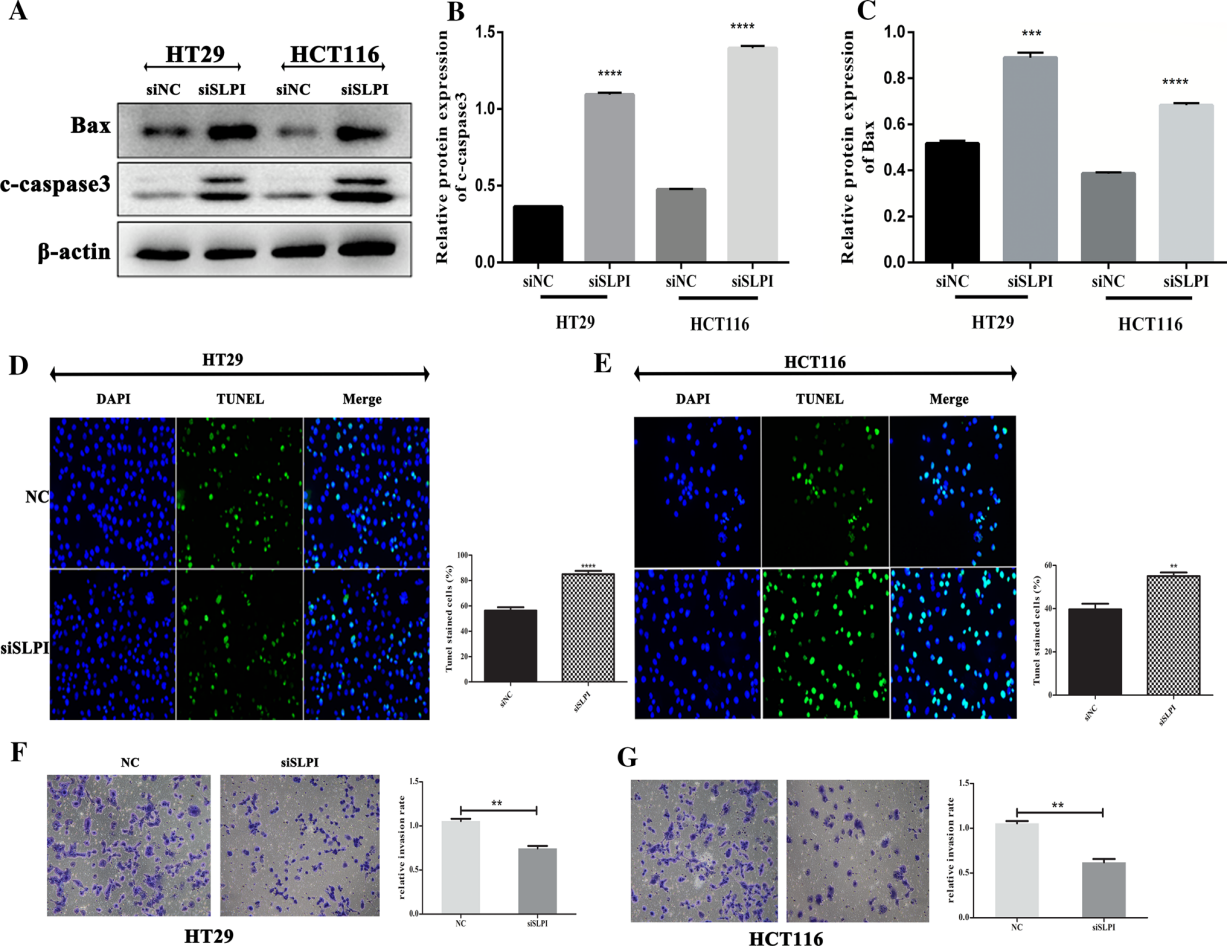
Puma can increase the level of apoptosis. Western blot results exhibited how inhibition of SLPI led to elevated expressions of both BAX protein and clavated-caspase 3 (**A**–**C**). The Tunel results confirmed the presence of significant apoptosis reaction in colon cancer cells in response to SLPI inhibition (**D**–**G**). Images were taken from three random groups, and TUNEL-positive cells were counted, and the percentage was calculated for HT29 and HCT116 cells. Tune-positive cells were found in calculated and *p < 0.05; ***p < 0.1, and ****p < 0.5

We performed the cell apoptosis assays to evaluate the synergic effect of SLPI inhibition and PUMA expression in colorectal cancer cells and assessed the expression pattern of c-caspase-3 and Bax, two essential apoptosis biomarkers. The western blotting results showed upgraded levels of BAX and c-caspase-3 protein expressions in CRCs in response to SLPI inhibition (Fig. 3A–C). Moreover, the TUNEL staining results exhibited advanced apoptotic reactions in HT-29 and HCT116 treated with siSLPI compared to the control (Fig. 3D, E). Together, these results confirmed that the aberrant expression of SLPI can suppress the expression of PUMA, block its pro-apoptotic functions, and subsequently promote tumor growth. Thus, suppressing the expression of SLPI in CRCs might be a potential path to promote colon cancer treatment. Furthermore, we performed transwell assays to visualize the cell invasion abilities of cells under siSLPI treatment. We found that the suppression of SLPI expression led to a lower rate of the invasion when compared to the control, confirming the essential role of SLPI protein in CRC development (Fig. 3F, G).

### Suppression of SLPI enhanced chemosensitivity of colon cancer cells by activating PUMA/BAX pathway in CRCs

**Fig. 4:**
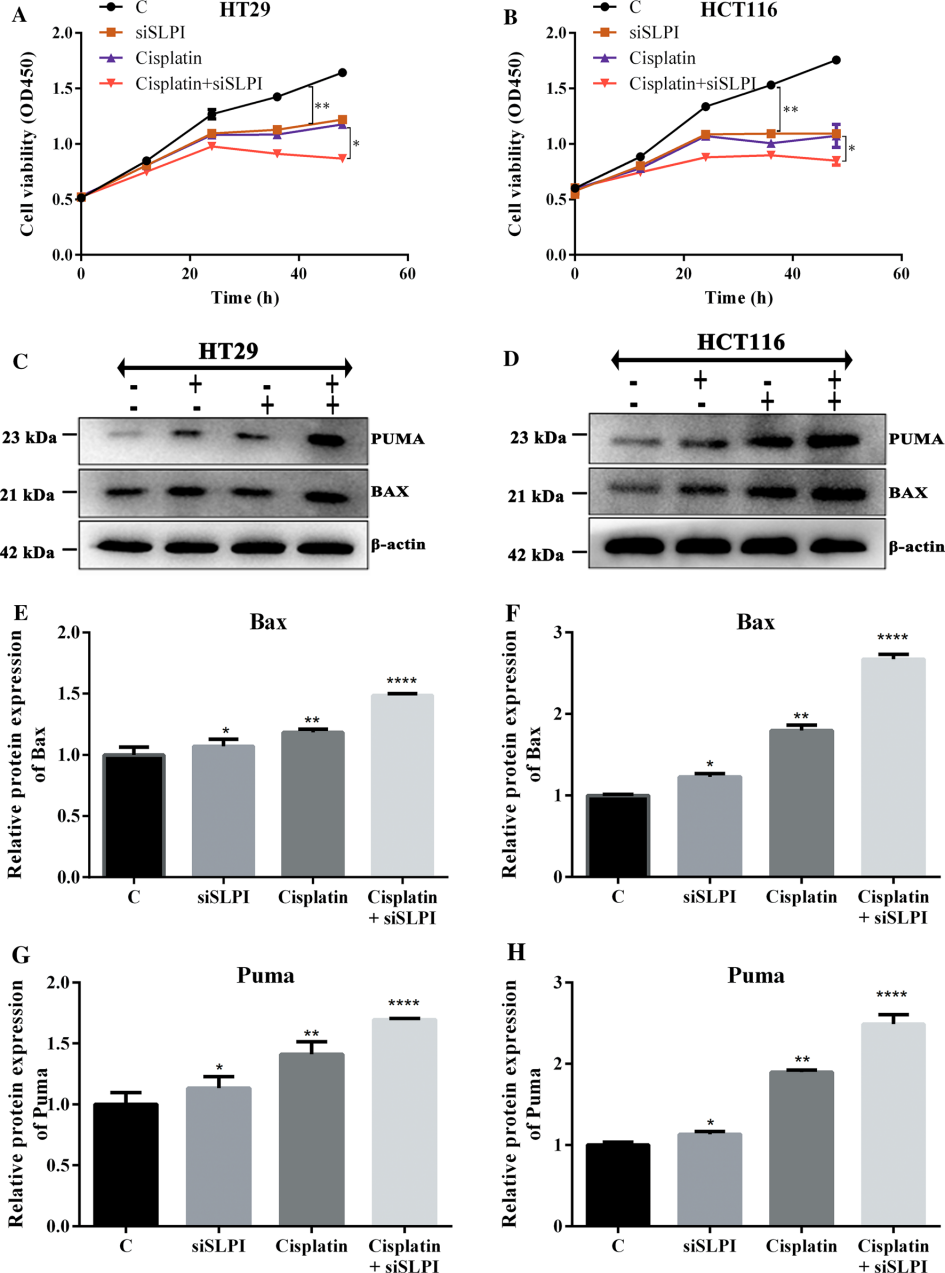
siSLPI enhanced chemosensitivity of colon cancer cells to Cisplatin (200 nm). Relative viability of cell treated siSLPI, Cisplatin and Cisplatin + siSLPI, the combined treatment of both Cisplatin and suppression of SLPI exhibited the lowest cell viability (**A**, **B**). Western Blot results showing how the suppression of SLPI coupled with cisplatin treatment induced higher expression levels of PUMA and BAX proteins than that of HT29 cells treated with Cisplatin or siSLPI alone (**C**, **D**). Scale bar representation of the protein expressions of PUMA and Bax respectively (**E**–**H**). *p < 0.05; **p < 0.1, and ****p < 0.5

To further assess the potential role of SLPI in modulating chemosensitivity of CRC cells, cells were infected with siSLPI and then treated for further 48 h with Cisplatin at the minimal dose. Using the two colon cancer cell lines, HT29 and HCT116, we explored the association between SLPI inhibition and cisplatin sensitivity in the following set of research. We monitored four types of treatment on CRCs, including the control with neither siSLPI nor Cisplatin (–) siSLPI groups without Cisplatin (+−) Cisplatin groups without siSLPI (−+) and siSLPI + Cisplatin group (++). After that, we evaluated the viability of both HT29 and HCT116 cells in the four groups. Our results revealed that all cells treated with combined Cisplatin and siSLPI (++) displayed the lowest cell viability rate compared with cells treated siSLPI or Cisplatin individually (*p < 0.05 versus the negative control group) (Fig. 4A, B). Western blotting analyses were then applied to assess the protein expressions of PUMA and Bax in both cell lines at all periods. Our study revealed that the expression levels of PUMA and Bax were the highest in cancer cells treated with both Cisplatin and siSLPI compared with other groups (Fig. 4C–H). The above revelations showed that SLPI suppression has similar effects as Cisplatin treatment of colon cancer cells.

### Suppression of SLPI enhanced cisplatin DDP therapy by improving the Caspase-3 induced apoptosis pathway in colon cancer cells in vivo

**Fig. 5:**
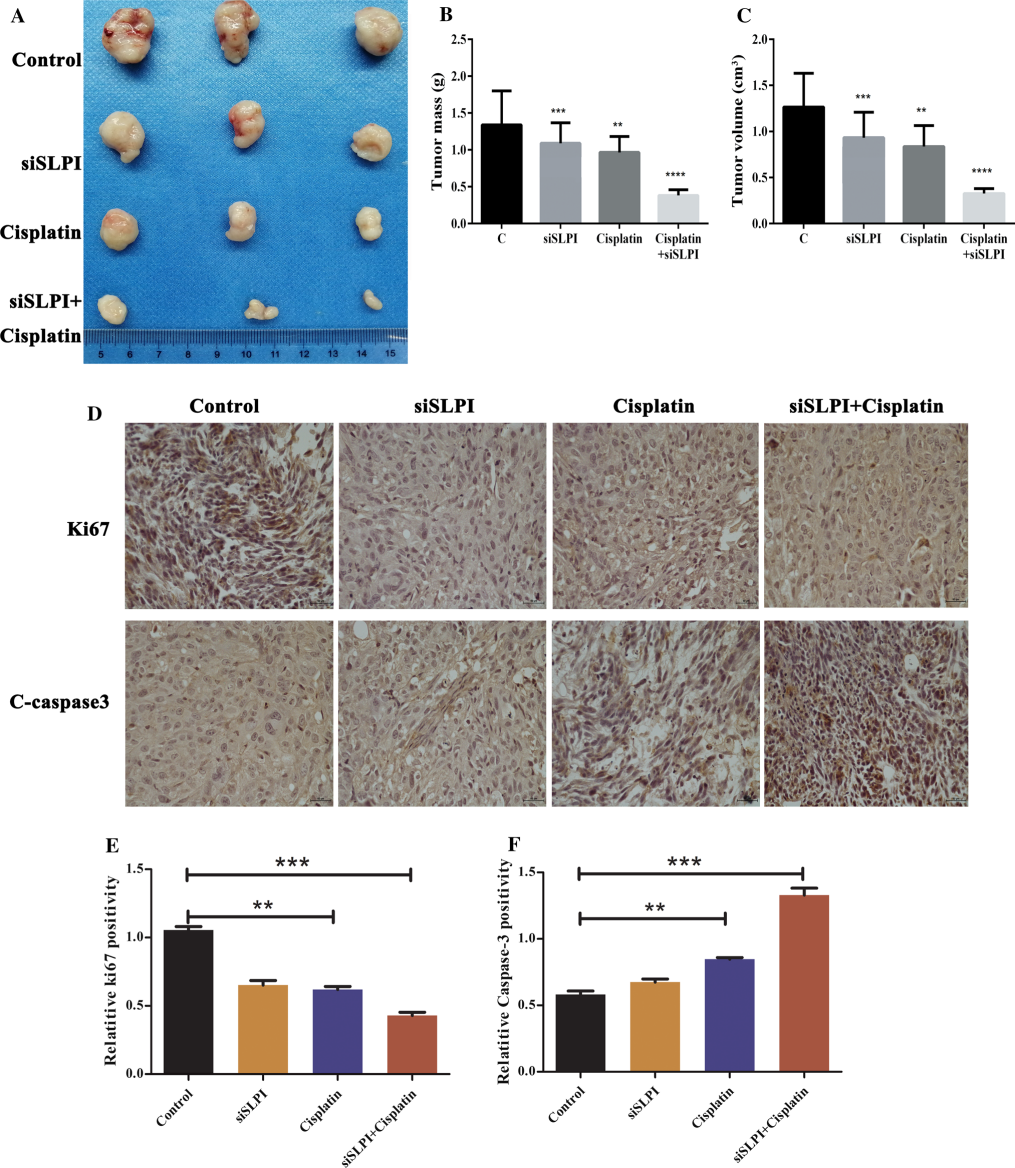
In vivo Effects of siSLPI and cisplatin tumor growth. The tumor sizes in the Cisplatin + siSLPI group was smaller than that in the Cisplatin + siSLPI groups (**A**). The combined treatment with Cisplatin + siSLPI displayed the lowest tumor weight (**B**). The tumor volume of Cisplatin plus siSLPI is considerably reduced (**C**). Immunohistochemical results showed that Ki67 level was downregulated while the c-caspase3 level was upregulated in the Cisplatin + siSLPI group (**D**–**F**) *p < 0.05; **p < 0.1, and ****p < 0.5

We further investigated the role of SLPI on the in vivo chemosensitivity of colon cancer cells by evaluating cell reaction to Cisplatin DDP, a famous chemotherapy drug used in many cancer treatments including the colorectal cancer notably [21]. BALB/C nude mice (6–8 weeks) were randomly selected and assigned to four treatments, including (–), (−+), (+−) and (++) groups. Therefore, we measured the tumor volume and observed their morphology to evaluate cancer evolution in different mice groups. The tumor volume in all treated groups (siSLPI group, cisplatin group, and cisplatin + siSLPI) significantly decreased compared to the control group. Meanwhile, the mice treated with Cisplatin + siSLPI exhibited the smallest tumor volume among all treated mice. In addition, the combination of Cisplatin and siSLPI generated the smallest tumor weights and masses, even though individual treatments with either Cisplatin or siSLPI induced similar but less significant effects (Fig. 5A–C). Finally, we monitored the Ki67 immunohistochemistry using the c-caspase-3 antibody to investigate tumor cell proliferation. As depicted in Fig. 5D–F, the Immunohistochemical results showed that the combination treatment (Cisplatin + siSLPI) exhibited the lowest level of Ki67, which is strictly associated with tumor growth, and the highest c-caspase-3 level an apoptosis biomarker. Therefore, we concluded that suppressing SLPI enhanced the cisplatin chemosensitivity or therapy by promoting cancer cell apoptosis in colon cancer.

### Tumour growth is associated with cytoplasmic expression of Akt and PUMA in CRC

**Fig. 6:**
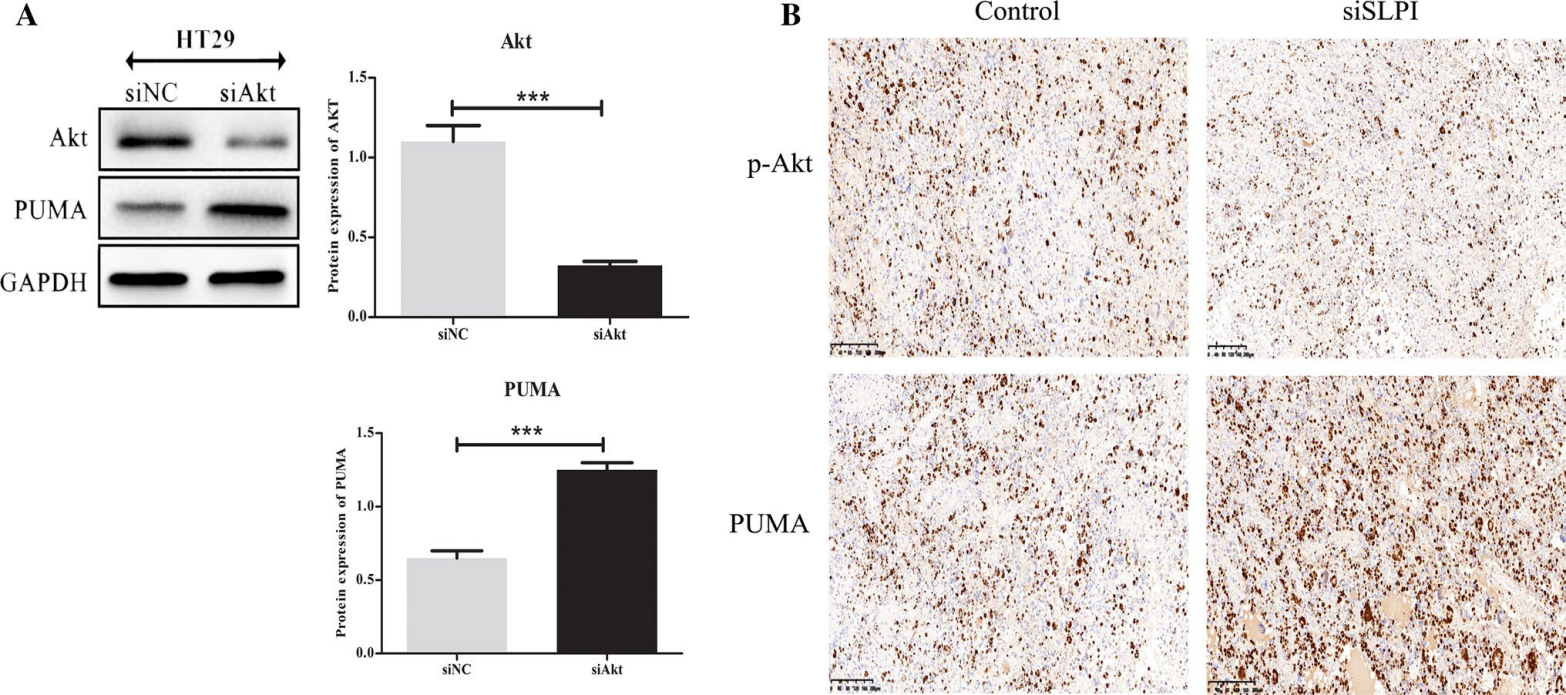
Tumour growth is associated with expression of Akt and PUMA in CRC Expression of Akt, PUMA and GAPDH (loading control) in HT-29 cells transfected with Akt-siRNA (**A**). IHC to measure PUMA and AKT in residual tumors after inhibition of SLPI in CRC (**B**). *p < 0.05; **p < 0.1, and ****p < 0.5

In a previous study, we demonstrated that the developments CRCs were significantly suppressed following the siRNA mediated AKT knockdown and suggested the AKT as a potential target for the treatment of colorectal cancers with high SLPI expression. Herein, we measured the PUMA expression in two both HT29 and HCT116 lines after AKT knockdown to demonstrated that the inhibition of SLPI repressed the AKT pathway to promotes PUMA expression in CRC (Fig. 6A). Many studies have mentioned the PUMA proteins among the most phosphorylation targets of Akt [22, 23]. The p-Akt stimulates the nuclear exclusion of important pro-apoptotic proteins like p27 and PUMA to promote the cellular proliferation. Interestingly, the proliferation of breast cancer cells was associated with cytoplasmic overexpression of p27 and Akt phosphorylation [23, 24]. In addition, the dual suppression of mTORC1 and mTORC2 expressions reduced the phosphorylation of Akt and generated Puma-dependent apoptosis in lymphoid malignancies Blood [25]. Therefore, we performed IHC to measure PUMA and AKT in residual tumor and found that the lowered activation of Akt was associated with enhanced cytoplasmic accumulation of PUMA (Fig. 6B). Thus, the inhibition of SLPI promotes PUMA expression by blocking the Akt pathway in CRCs.

## Discussion

First discovered in patients who underwent a chronic obstructive pulmonary disease (COPD) and later linked with COPD prevalence and other cancers like pancreatic ductal adenocarcinoma, SLPI has been gaining more attention in cancer research [26, 27]. We previously demonstrated that silencing of SLPI through siRNA promoted cell apoptosis and stopped the progression and invasion of pancreatic ductal adenocarcinoma cells. A recent study reported that the SLPI knockdown prevents the migration of colon cancer cells in vitro through the modulation of Akt signaling [28].

Here, we showed that the inhibition of SLPI in HT-29 and HCT116 colorectal cancer cell lines resulted in a remarkable increase of PUMA protein expression and consequently reduced the cancer cell viability (Fig. 1). This section suggested that the SLPI gene might play an essential role in human colon cancer cells by regulating PUMA-dependent reactions. In addition, besides enhancing PUMA expressions, the SLPI inhibition modified the phosphorylation of Akt, fox3a, and p65, synchronously. It is important to note that the nucleolar accumulation of p65 modulated the NF-κB-driven transcription and apoptosis [29]. Hence, the increased concentration of Akt and p-p65 in the colorectal cancer cell in response to SLPI suppression suggested that SLPI genes regulated CRC cell apoptosis through Akt/NF-κB/PUMA signaling pathways (Fig. 2). The caspase-3 represented a valuable cell apoptosis biomarker for numerous carcinomas like cervical adenocarcinoma, colorectal cancer, and glioma [30]. The colon cell apoptosis, characterized through western blot and TUNEL staining assays, exhibited upgraded expressions of caspase-3 and Bax proteins in response to SLPI inhibition (Fig. 3). Thus, the suppression of SLPI enhanced CRCs apoptosis through activation of Akt/NF-κB/PUMA signaling.

Moreover, recent studies revealed that the PUMA adenovirus promoted drug sensitivity in oesophageal and ovarian cancers. In addition, the p53/miR-503-5p/PUMA signaling pathway modulated the CRC chemosensitivity and hypnotized that miR-503-5p regulated the development of CRC by regulating PUMA expression [26, 27].

Herein, we assessed the half-maximal inhibitory concentration (IC50) of cisplatin in SLPI-siRNA transfected CRCs using the non-transfected cells as controls. HT29 and HCT116 cells were treated with different doses of cisplatin, and cell survival was evaluated using the MTT assay. As a result, the survival rate of SLPI siRNA-transfected cells did not differ significantly from that of the negative control siRNA-transfected and untreated cells at a 7.6 µg/mL cisplatin dosage (Additional file 1: Fig. S1). Meanwhile, the IC50 concentration of cisplatin for cancer cells reduced dramatically from 5.8 µg/mL in control cells to 2.27 µg/mL in SLPI siRNA-transfected cells, demonstrating that siSLPI enhances the CRC chemosensitivity to Cisplatin treatment. The combined treatments with siSLPI and Cisplatin induced a much lower cell viability rate than treatment with either siSLPI or cisplatin used independently, confirming that the inhibition of SLPI increased chemosensitivity to Cisplatin in CRC. The highest expression level of PUMA in colon cancer cells treated with Cisplatin and siSLPI was associated with the lowest cell proliferation rate (Fig. 4). Hence, the sensitivity of the colon cancer cells to Cisplatin is strictly linked to SLPI and PUMA expressions. Importantly, the tumor volume in BALB/C nude mice treated with siSLPI, Cisplatin, and Cisplatin + siSLPI significantly decreased. Notably, the tumor volume of the Cisplatin + siSLPI group was smallest than other groups. Our results evidenced that the combination of Cisplatin and siSLPI minimized the tumor volume and weight. Finally, the lowest level of Ki67 associated with the highest level of c-caspase-3 exhibited by cisplatin + siSLPI groups during the in vivo Immunohistochemical analysis justified that SLPI proteins play crucial roles in regulating cisplatin chemosensitivity of colon cancer cells (Fig. 5).

In addition, the IHC results of PUMA and AKT expression in residual tumors confirmed the inhibition of SLPI promotes PUMA expression by blocking the Akt pathway in CRCs. Thus PUMA (Fig. 6B). Thus, our results unveiled a novel approach to reduce CRC death and to promote the development of more efficient CRC-cisplatin drug treatment.

## Conclusion

In sum, our study confirmed that the suppression of SLPI effectively improved the protein expression of the PUMA and promoted the PUMA-mediated CRC cells’ apoptosis consequently. In addition, it highlights the interaction between the SLPI knockdown and critical biological factors, such as phosphorylated Akt, FoxO3, and p65, c-caspase-3. More importantly, we demonstrated that suppressing SLPI can enhance the chemosensitivity of colorectal cancer cells the Cisplatin by activating the Akt/PUMA signaling pathways. Thus, our findings confirm the pivotal role of SLPI in the development of colon cancer and its potential status as a target for therapeutics research.

## Supplementary Information

Additional file 1 Figure S1. Half-maximal inhibitory concentration (IC50) of cisplatin in SLPI-siRNA transfected CRCs using the non-transfected cells as controls. The IC50 concentration of cisplatin for CRC reduced dramatically from 5.8 µg/mL in control cells to 2.27  µ g/mL in SLPI siRNA-transfected cells. (TIF 21504 KB)

## Data Availability

The datasets used and/or analyzed during the current study are available from the corresponding author on reasonable request.

## Abbreviations

BAX: Bcl-2-associated X protein
BCL-2: B-cell lymphoma-2
CRC: Colorectal cancer
LncRNA: Long noncoding RNA
SLPI: Inhibition of Secretory Leukocyte Protease Inhibitor
PUMA: P53-up-regulated modulator of apoptosis
Akt: Protein kinase B
NF-κB: NF-kappaB, nuclear factor kappa-light-chain-enhancer of activated B cells
