# Supplementary figures and images for: Inhibition of secretory leukocyte protease inhibitor (SLPI) promotes the PUMA-mediated apoptosis and chemosensitivity to cisplatin in colorectal cancer cells
Source: Discov Oncol. 2023 Jan 3;14:1. doi: 10.1007/s12672-022-00535-9 (PMC9810770; doi:10.1007/s12672-022-00535-9)

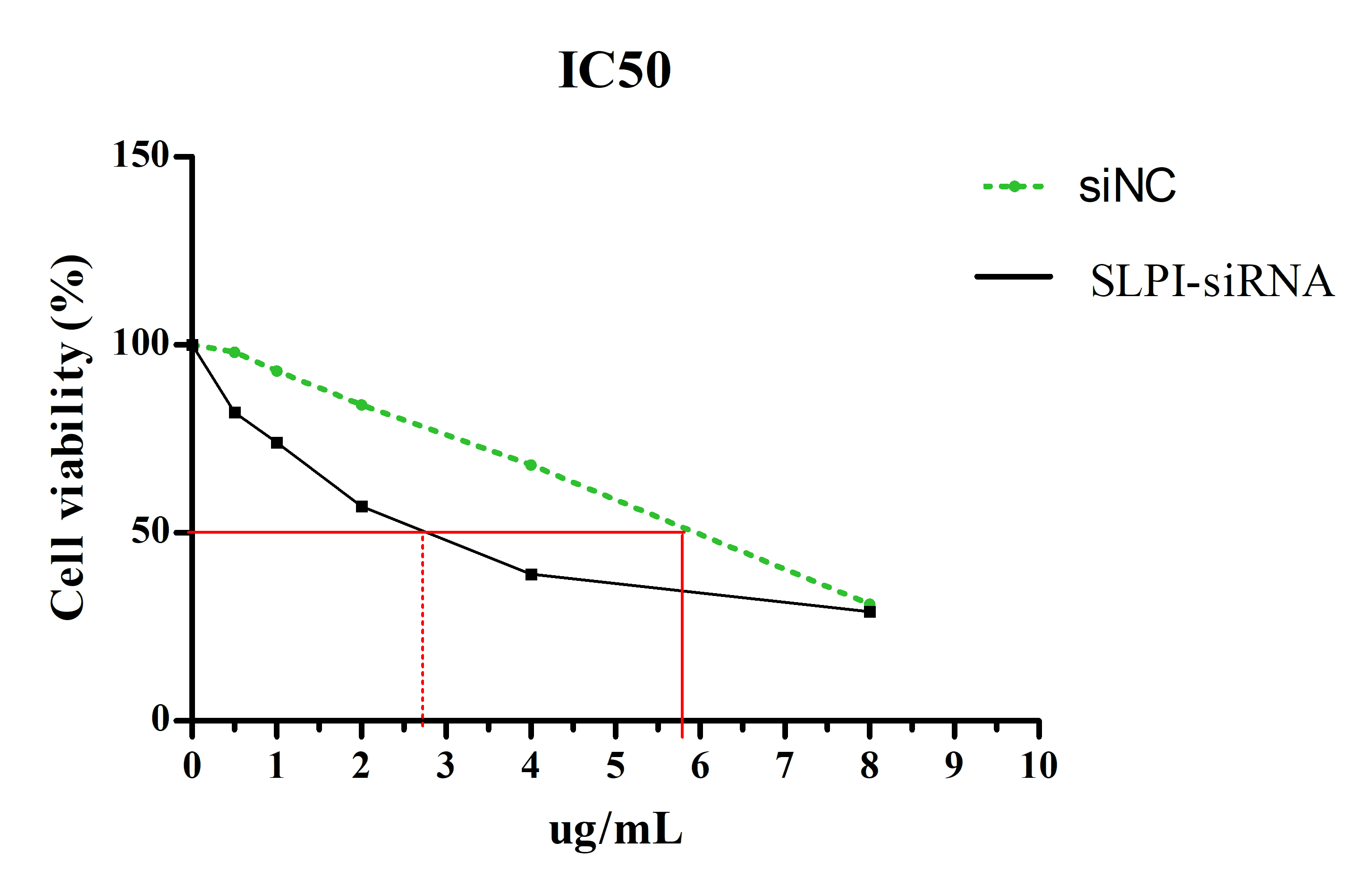

Supplement: Supplementary file 1 — Additional file 1 Figure S1. Half-maximal inhibitory concentration (IC50) of cisplatin in SLPI-siRNA transfected CRCs using the non-transfected cells as controls. The IC50 concentration of cisplatin for CRC reduced dramatically from 5.8 µg/mL in control cells to 2.27 µ g/mL in SLPI siRNA-transfected cells. (TIF 21504 KB) [file 12672_2022_535_MOESM1_ESM.tif]
